# Incidence of Potential Drug-Drug Interaction and Related Factors in Hospitalized Neurological Patients in two Iranian Teaching Hospitals

**Published:** 2014-11

**Authors:** Soha Namazi, Shiva Pourhatami, Afshin Borhani-Haghighi, Sareh Roosta

**Affiliations:** 1 Health Policy Research Center, Department of Pharmacotherapy, School of Pharmacy, Shiraz University of Medical Sciences, Shiraz, Iran;; 2 Research Center for Traditional Medicine, and History of Medicine, Department of Pharmacotherapy, School of Pharmacy, Shiraz University of Medical Sciences, Shiraz, Iran;; 3 Clinical Neurology Research Center, Department of Neurology, Shiraz University of Medical Sciences, Shiraz, Iran;; 4 Vice Chancellery of Research and Technology, Center for Development of Clinical Studies, Nemazee Hospital, Shiraz University of Medical Sciences, Shiraz, Iran

**Keywords:** Drug-related side effects and adverse reactions, Neurology, Risk factor

## Abstract

**Background: **Reciprocal drug interactions are among the most common causes of adverse drug reactions. We investigated the incidence and related risk factors associated with mutual drug interactions in relation to prescriptions written in the neurology wards of two major teaching hospitals in Shiraz, southern Iran.

**Methods: **Data was collected from hand-written prescriptions on a daily basis. Mutual drug interactions were identified using Lexi-Comp 2012 version 1.9.1. Type D and X drug interactions were considered as potential drug-drug interactions. The potential risk factors associated with drug-drug interactions included the patient’s age and gender, number of medications and orders, length of hospitalization and the type of neurological disorder. To determine potential drug-drug interactions, relevant interventions were suggested to the physicians or nurses and the outcome of the interventions were documented.

**Results: **The study comprised 589 patients, of which 53% were males and 47% females, with a mean age of 56.65±18.19 SD years. A total of 4942 drug orders and 3784 medications were prescribed among which 4539 drug-drug interactions were detected, including 4118 type C, 403 type D, and 18 type X. Using a logistic regression model, the number of medications, length of hospitalization and non-vascular type of the neurological disorder were found to be significantly associated with potential drug-drug interactions. From the total interventions, 74.24% were accepted by physicians and nurses.

**Conclusion:** Potentially hazardous reciprocal drug interactions are common among patients in neurology wards. Clinical pharmacists can play a critical role in the prevention of drug-drug interactions in hospitalized patients.

## Introduction

The concept of medication errors (MEs) and adverse drug events (ADEs) have received extensive attention among the public and the medical community in recent decades.^1^ According to a report published by the Institute of Medicine, annually 98000 deaths due to MEs occur in hospitals.^2^ The cost of drug-related morbidity and mortality is expected to be 76.6 billion dollars per year in the United States.^3^ Potential drug-drug Interactions (PDDIs) are preventable and are common causes of ADEs.^4^^-^^8^ PDDIs require intervention protocol including administration of appropriate alternative medicine, dose adjustment and monitoring clinical signs and symptoms of ADEs by health care professionals to minimize possible ADEs.^9^^-^^11^

Neurological illnesses are among the most common causes of hospitalization in Iran.^12^ Therefore; ADEs can be prevented by assessment and determination of drug-drug interaction (DDIs) in neurology wards, and reduce the length and cost of hospitalization.

To the best of our knowledge, there is no published report on DDIs in neurological disorders in Iran or other countries, however similar studies in other hospital wards has been done.^5^^-^^8^ The present study was instigated to determine the incidence of DDIs in patients admitted to neurology wards and to identify PDDIs risk factors.

## Materials and Methods

This cross sectional study conducted in the neurology wards of Nemazee and Faghihi hospitals (Shiraz, Iran) from March to September 2012. These referral hospitals are affiliated with Shiraz University of Medical Sciences, which admit all patients from the southern part of Iran without limitation. Due to the lack of Computerized Physician Order Entry (CPOE), automatic determination of DDIs was not possible. In these wards, drug ordering and administration are mainly handwritten. Physicians prescribe drug orders on patients’ files and then nurses transcribed them on administration charts. Standard practice at these wards includes daily rounds by the attending neurologist to recommend drugs, accompanied by neurology residents to document drug orders. In this study, a pharmacy student reviewed patients’ files, laboratory data, and physician orders on a daily basis. Information regarding the patients and physicians were treated as confidential.

The inclusion criteria were patients admitted to the neurology ward and receiving at least two medications. Initially, demographic information of patients such as age, gender, and clinical diagnosis were recorded. Neurological disorders were classified into vascular and non-vascular types. The vascular type comprised all neurological disorders due to pathologies in cerebral and spinal arteries, capillary, and veins. Non-vascular group contained all other neurological disorders due to degenerative, inflammatory/infectious, demyelinative/immunologic, toxic-metabolic, traumatic, developmental, congenital, environmental, neoplastic, epileptic, cephalalgia and other pathologies. 

The prescribed medications and doses, intervals and length of drug use were recorded in a document designed for this purpose. All prescribed medications were divided into eight categories according to the “Drug Facts and Comparisons 2009” manual. ^13^Primary source for identifying DDIs was Lexi-Comp version 1.9.1^14^ which was used for classifying DDIs into the following five groups.^14^

A: There are no pharmacodynamic or pharmacokinetic interactions with the concurrent administration of two drugs.
B: May interact with each other, but there is no clinically significant interaction, no action is required.
C: The benefits of co-administration usually outweigh the harm, the patient should be monitored.
D: There is a strong interaction between the two drugs. Intervention should take place, frequency of use or amount of drugs should be changed or if possible use alternative medicine.
X: The harm related to concomitant administration outweigh the benefits, co-administration is prohibited or contraindicated.

The software provided information such as drug class, type, severity, and reliability of interaction, management, and intervention. Type C, D and X DDIs were recorded for all recruited patients. Type D and X DDIs were considered as PDDI.^15^ For a better evaluation, DDIs were also divided into two categories including 1-DDI which received at least one drug affecting CNS (neurological drugs), and 2-DDI indicating interaction between drugs with no effect on the CNS (non-neurological drug). Following the identification of DDI category, relevant interventions were recommended to the residents or nurses by the research assistant. Interventions were divided into two categories. The first included administrative interventions carried out by nurses that administered medications and related to those DDIs in which medications interacted with each other during consumption, such as Ciprofloxacin and divalent cations. The second was prescription interventions conducted by residents that referred to the interactions between medications prescribed by physicians, such as concurrent administration of Omeprazole and Clopidogrel. All patients were monitored and the outcome of DDIs/PDDIs and the ADEs attributed to DDIs/PDDIs were recorded throughout the hospitalization period.

*Statistical Analysis*

Statistical analysis was conducted using SPSS version 15. The continuous data was expressed as mean±SD and the categorical data reported as a percent or frequency. The *t*-test was used to compare means of quantitative variables and Chi-square test applied to compare qualitative variables. The correlation between age and PDDIs was investigated by Pearson correlation coefficient test. The same test was used to evaluate the relationship between each of the variables consisting of order quantity, medications and length of hospitalization and the rate of PDDIs. The association between neurological disorders and PDDIs was determined by Chi-square test. The simultaneous impact of all affective variables on PDDIs was investigated by logistic regression, which determined variable’s odds ratio and 95% confidence interval. P value less than 0.05 was considered as significant.

## Results

The study comprised 600 patients, of which 11 cases were excluded because of receiving less than two medications during hospitalization. The remaining 589 patients were included in this study. Among these, 354 (60.10%) patients were in Nemazee and 235 (39.90%) patients in Faghihi hospitals. Table 1 shows demographic and clinical characteristics of the recruited patients. The mean of prescribed medication for each patient was 6.58±3.41. Vascular disorders were the most common cause (58.57%) of hospitalization among our patients.

**Table 1:** Demographic and clinical characteristics of patients (N=589)

**Age (year)**	**Range**	**(15-94)**
	**Mean±SD**	**56.65±18.19**
Sex N (%)	Male (%)	312 (53.00%)
	Female (%)	277 (47.00%)
Length of hospitalization (days)	Range	(2-45)
	Mean±SD	6.67±4.38
Number of prescribed drugs	Range	(1-21)
	Mean±SD	6.58±3.41
Number of drug orders	Range	(3-50)
	Mean±SD	8.39±5.21
Clinical diagnosis N (%)	Vascular	345 (58.57%)
	Non Vascular	244 (41.43)

During the study period, 3748 medications were prescribed. These included, CNS drugs (N=973, 25.96%), cardiovascular drugs (N=969, 25.85%), gastrointestinal drugs (N=885, 23.61%), supplements (N=360, 9.61%), systemic antibiotic drugs (N=340, 9.07%), biologic and immunologic drugs (N=118, 3.15%), endocrine and metabolic drugs (N=76, 2.03%) and renal and genitourinary (N=27, 0.72%). The most prescribed medications were Ranitidine (N=352, 58.97%), Atorvastatin (N=314, 52.30%), Heparin (N=243, 40.50%), Aspirin (N=235, 39.20%) and Clopidogrel (N=117, 19.50%).

A total of 4539 DDIs was detected. Table 2 shows the incidence of different types of DDIs presented as C, D, and X as well as neurologic and non-neurologic in the recruited patients. The most common C, D, and X DDI were found between Heparin-Aspirin (23.40%), Warfarin-Aspirin (16.30%), and Omeprazole-Clopidogrel (0.60%). Due to the limited number and importance of type X DDIs, these are demonstrated in table 3.

**Table 2:** The distribution of different types of DDIs* (N=589)

Type C DDI	Total number of C DDI				4118
	Mean±SD/Patient, (Minimum, Maximum) (0-55)				8.23±6.99
Neurologic	Number (%)			2143 (52.04)
Non Neurologic	Number (%)			1975 (47.96)
Type D DDI	Total Number of D DDI				403
	Mean±SD/Patient, (Minimum, Maximum) (0-10)				1.29±0.68
	Neurologic			Number (%)	257 (63.77)
	Non Neurologic			Number (%)	146 (36.23)
Type X DDI	Total Number of X DDI				18
	Mean±SD/Patient, (Minimum, Maximum) (0-2)				0.19±0.03
	Neurologic		Number (%)		13 (72.22)
	Non Neurologic		Number (%)		5 (27.78)

*DDI: Drug-Drug interaction

**Table 3:** The most frequent type X DDIs* (N=18)

**X DDI**		Mechanism of interaction**
**Drugs with interactions**	**Number (%)**	
Clopidogrel-Omeprazole	3 (0.60%)	The proposed mechanism is Omeprazole inhibition of the CYP450 2C19-mediated metabolic bioactivation of clopidogrel.
Chlorpromazine- Metoclopramide	2 (0.40%)	Co-administration may increase the frequency and severity of extrapyramidal reactions (i.e. acute dystonic reactions, tardive dyskinesia, akathisia, Parkinson-like symptoms) due to additive antidopaminergic effects.
Sertraline-Clopidogrel	2 (0.40%)	Sertraline (CYP450 2C19 inhibitor) may decrease serum concentration of active metabolites of Clopidogrel.
Clopidogrel –Fluoxetine	2 (0.40%)	Fluoxetine (CYP450 2C19 inhibitor) may decrease serum concentration of active metabolites of Clopidogrel.
Diazepam-Olanzapine	2 (0.40%)	Olanzapine may enhance the adverse effect of Benzodiazepine (Cardiorespiratory depression, excessive sedation)
Sucralfate-Calcitriol	1 (0.20%)	Calcitriol may increase the serum concentration of Sucralfate. Specifically, the absorption of aluminum from Sucralfate may be increased.
Olanzapine-Alprazolam	1 (0.20%)	Olanzapine may enhance the adverse effect of Benzodiazepine (Cardiorespiratory depression, excessive sedation)
Thioridazine-Doxepine	1 (0.20%)	Co-administration can cause prolongation of the QT interval, and may result in elevated risk of ventricular arrhythmias including ventricular tachycardia and torsade de pointes
Citalopram-Tetrabenazine	1 (0.20%)	Co-administration can cause prolongation of the QT interval, and may result in elevated risk of ventricular arrhythmias including ventricular tachycardia and torsade de pointes
Clonazepam-Olanzapine	1 (0.20%)	Olanzapine may enhance the adverse effect of Benzodiazepine (Cardiorespiratory depression, excessive sedation)
Tizanidine-Ciprofloxacin	1 (0.20%)	The proposed mechanism is ciprofloxacin inhibition of tizanidine metabolism via CYP450 1A2 and may significantly increase the plasma concentrations and pharmacologic effects of tizanidine.
Thioridazine-Maprotiline	1 (0.20%)	Co-administration can cause prolongation of the QT interval, and may result in elevated risk of ventricular arrhythmias including ventricular tachycardia and torsade de pointes

*Drug-Drug interaction; **This is based on Lexi-comp 2013^14^

A total of 484 ADEs was found. The most common ADEs caused by DDIs were platelet and clotting disorder (31.82%), cardiovascular disorder (28.10%), liver and biliary system disorder (15.08%), metabolic and nutritional disorder (14.26%), as well as urinary system disorder (10.74%).

The gender of the patients did not have a significant effect on PDDIs (P=0.44). We also found no correlation between age and PDDIs (Pearson correlation coefficient=0.08, P=0.23). However, a significant relationship was found between the incidence of PDDIs and neurological disorders (P<0.001).

The *t* test showed that the mean±SD of the number of orders in patients with at least one PDDI was significantly higher than those without any PDDIs (10.6±6.53 *vs.* 7.17±3.80, P<0.001). This was also true about the number of prescribed medications (8.90±3.40 *vs.* 5.30±2.65, P<0.001), and the length of hospitalization (8.26±5.65 days *vs.* 5.80±3.18, P<0.001).

Logistic regression was used to investigate the impact of sex, age, the length of hospital stay, number of medications, orders and neurological disorder on PDDIs (table 4). The frequency of PDDIs increased significantly by non-vascular disease, number of prescribed drugs and length of hospitalization. The risk of incidence of PDDI in patients receiving more than five drugs was 6.91 times higher than those receiving less than five drugs (P<0.001, 95% Confidence Interval=4.23-11.27). Non-vascular disorders increased the risk of PDDIs by 1.64 times (95% Confidence Interval=1.03-2.63) compared with vascular disorders. Patients with more than five days hospitalization experienced PDDIs 1.75 times higher compared with other patients. In the presence of DDI, appropriate interventions were unofficially recommended by the research assistant. Among 373 prescription interventions 240 (64.34%) were accepted by the physicians and of 48 administrative interventions 28 (58.33%) were accepted by nurses. In general, among all interventions 74.24% were accepted by both physicians and nurses.

**Table 4:** Logistic Regression Analyzes of DDI* Data

**Variables**	**Categorial** ** Level**	**Odds ratio**	**95% confidence interval for odds ratio**	
			**Lower**	**Upper**
Patient age	15-30	Reference	-	-
	31-45	0.86	0.40	1.88
	46-60	0.70	0.33	1.50
	61-75	0.53	0.24	1.16
	>75	0.62	0.27	1.42
Number of drugs per prescription	<5	Reference	ـ	ـ
	>5	6.91	4.24	11.27
Gender	Female	Reference	ـ	ـ
	Male	1.22	0.83	1.81
Length of hospital stay	<5	Reference	ـ	ـ
	>5	1.75	1.11	2.78
Number of orders	<5	Reference	ـ	ـ
	>5	1.73	0.93	3.23
Type of neurological disease	Vascular	Reference	-	-
	Non Vascular	1.64	1.03	2.63

*Drug-Drug interaction

## Discussion

In this study, 35.5% of patients experienced at least one PDDI. This rate ranged from 17 to 72.5%, according to different study design, study population (general vs. ICU wards, and medical vs. surgical wards), definition of DDI, inclusion and exclusion criteria, and availability of clinical pharmacy services in previous studies.^15^^-^^21^

In a similar study conducted in Tehran-Iran, 203 post-CCU patients were evaluated for DDIs.^7^ 3166 PDDIs were recorded for which 75%, 4.71% and 1.73% were categorized as C, D and X DDIs respectively. In this study, mean number of drugs per patient was 11.22±3.91.^7^

It is found that the number of prescribed medications, non-vascular neurological disorders and length of hospitalization were the only attributing risk factors for the incidence of PDDIs, as determined by the logistic regression model. The number of orders was associated with PDDIs in univariate regression model. Since the number of orders might be expected to associate with the number of prescribed medications and length of hospitalization, Multivariate Regression Analysis showed no significant association between the incidence of PDDIs and such entity. The hazard of the increasing number of prescribed medications has been reported in previous studies.^21^ Meanwhile, PDDIs are most probably occur among inpatients since the number of prescribed drugs increased in hospital settings.^7^ Mannesse et al. reported the concurrent use of three or more drugs increases the risk of ADEs by 9.8 times.^21^ In another study, a linear relationship between the number of drugs per prescription and the frequency of interactions were found.^22^

In the current study, administration of more than five drugs increased the incidence of PDDIs by 6.90 times. Therefore, physicians and pharmacists should monitor the patients who concurrently receive multiple drugs in order to decrease the incidence of DDIs. Non-vascular disorders such as epilepsy increased the risk of PDDI compared with vascular disorders. More than 35% of our patients received at least one Antiepileptic drug. These drugs were considered as a risk factor for PDDI in previous studies.^23^^,^^24^ The risk of PDDIs did not increase with gender and age which is in contrast with previous studies.^15^^,^^18^^,^^25^^,^^26^ Elderly patients are probably more susceptible to PDDIs because of attendant co-morbidities. Such discrepancy can be explained in terms of the population considered in this study. They were only admitted to a particular ward of the hospital where 64.5% of the patients were above 50 years of age. In a more heterogeneous study population, the age factor could influence PDDIs.

Our study determined PDDIs outcome that was identified by reviewing clinical evidences and laboratory data. It is worth noting that the evaluation of PDDIs outcome requires patient’s specific assessment with a prospect of being clinically relevant and require the ability to monitor DDIs. Consequently, it is premature to discuss ADEs with certainty.

The prescription and administrative interventions, which were acknowledged by the health care team, were 62.81% and 58.33% respectively. According to a study by Reimche et al., the rate of PDDI in patients admitted to Ottawa hospital was 19.3%.^15^ In their study, clinical pharmacy services were provided during the study period and clinical pharmacists monitored the prescribed medications to patients. However, in our study, clinical pharmacists played no role in drug prescriptions and interventions were informally recommended by our research assistant. The interaction between clinician and pharmacist can be improved by the full-time presence of a clinical pharmacist in neurology wards. Since pharmacists are familiar with the devised PDDIs detection software, their presence during hospital rounds can guarantee a better care for patients. Such presence is specifically beneficial in wards such as neurology ward where multiple medications are prescribed.^27^

This study had certain limitations. Firstly, this investigation was only conducted in the neurology ward of two teaching hospitals where patients with certain disorders and particular medications are admitted. This negatively impacts the generalization aspect of the obtained results. Secondly, only one particular drug interaction software was used while some DDIs are detectable by other software packages. Thirdly, “pro re nata” (PRN) order was not included, which may result in underestimation of DDI rate. Finally, our patients were only monitored during hospitalization. This means that ADEs occurring after hospital discharge could be detected. 

## Conclusion

Potentially hazardous DDIs are common among hospitalized patients in the neurology ward in our hospitals, especially among those receiving multiple medications. Clinical pharmacists along with other health care professionals can play an essential role in the prevention, management of PDDIs and improvement of medication therapy in hospitalized patients. 
